# Acetyl-L-Carnitine downregulates invasion (CXCR4/CXCL12, MMP-9) and angiogenesis (VEGF, CXCL8) pathways in prostate cancer cells: rationale for prevention and interception strategies

**DOI:** 10.1186/s13046-019-1461-z

**Published:** 2019-11-12

**Authors:** Denisa Baci, Antonino Bruno, Caterina Cascini, Matteo Gallazzi, Lorenzo Mortara, Fausto Sessa, Giuseppe Pelosi, Adriana Albini, Douglas M. Noonan

**Affiliations:** 1 School of Medicine and Surgery, University of Milano-Bicocca, Building U8, Via Cadore 48, 20900 Monza, Italy; 2 Science and Technology Pole (PST), IRCCS MultiMedica, Milan, Italy; 3 Department of Biotechnology and Life Sciences, University of Insubria, Varese, Italy; 4 Department of Medicine and Surgery, University of Insubria, Varese, Italy; 5 Department of Oncology and Hemato-Oncology, University of Milan, Milan, Italy

**Keywords:** Carnitine, Prostate cancer, Angiogenesis, Inflammation, Angioprevention, Chemoprevention, Interception

## Abstract

**Background:**

Prostate cancer (PCa) is a leading cause of cancer-related death in males worldwide. Exacerbated inflammation and angiogenesis have been largely demonstrated to contribute to PCa progression. Diverse naturally occurring compounds and dietary supplements are endowed with anti-oxidant, anti-inflammatory and anti-angiogenic activities, representing valid compounds to target the aberrant cytokine/chemokine production governing PCa progression and angiogenesis, in a chemopreventive setting. Using mass spectrometry analysis on serum samples of prostate cancer patients, we have previously found higher levels of carnitines in non-cancer individuals, suggesting a protective role. Here we investigated the ability of Acetyl-L-carnitine (ALCAR) to interfere with key functional properties of prostate cancer progression and angiogenesis in vitro and in vivo and identified target molecules modulated by ALCAR.

**Methods:**

The chemopreventive/angiopreventive activities ALCAR were investigated in vitro on four different prostate cancer (PCa) cell lines (PC-3, DU-145, LNCaP, 22Rv1) and a benign prostatic hyperplasia (BPH) cell line. The effects of ALCAR on the induction of apoptosis and cell cycle arrest were investigated by flow cytometry (FC). Functional analysis of cell adhesion, migration and invasion (Boyden chambers) were performed. ALCAR modulation of surface antigen receptor (chemokines) and intracellular cytokine production was assessed by FC. The release of pro-angiogenic factors was detected by a multiplex immunoassay. The effects of ALCAR on PCa cell growth in vivo was investigated using tumour xenografts.

**Results:**

We found that ALCAR reduces cell proliferation, induces apoptosis, hinders the production of pro inflammatory cytokines (TNF-α and IFN-γ) and of chemokines CCL2, CXCL12 and receptor CXCR4 involved in the chemotactic axis and impairs the adhesion, migration and invasion capabilities of PCa and BPH cells in vitro. ALCAR exerts angiopreventive activities on PCa by reducing production/release of pro angiogenic factors (VEGF, CXCL8, CCL2, angiogenin) and metalloprotease MMP-9. Exposure of endothelial cells to conditioned media from PCa cells, pre-treated with ALCAR, inhibited the expression of CXCR4, CXCR1, CXCR2 and CCR2 compared to those from untreated cells. Oral administration (drinking water) of ALCAR to mice xenografted with two different PCa cell lines, resulted in reduced tumour cell growth in vivo.

**Conclusions:**

Our results highlight the capability of ALCAR to down-modulate growth, adhesion, migration and invasion of prostate cancer cells, by reducing the production of several crucial chemokines, cytokines and MMP9. ALCAR is a widely diffused dietary supplements and our findings provide a rational for studying ALCAR as a possible molecule for chemoprevention approaches in subjects at high risk to develop prostate cancer. We propose ALCAR as a new possible “repurposed agent’ for cancer prevention and interception, similar to aspirin, metformin or beta-blockers.

## Background

Prostate cancer (PCa) represents the most commonly diagnosed malignancy in men and the second cause of male cancer death worldwide [1, 2]. Substantial evidence suggests that chronic inflammation and angiogenesis contribute to tumour initiation, metastasis, and progression [3–6]. The inflammatory infiltrate has been reported to support the development of PCa [7–9]. Prospective studies found that PCa patients with greater extent of intra prostatic inflammation exhibit poorer outcome [10, 11].

Diverse naturally occurring compounds and dietary supplements (such as polyphenols, flavonoids, carotenoids etc) have been reported to be endowed with chemopreventive and angiopreventive activities in PCa [12, 13], by targeting multiple pathways, thus interfering with cancer insurgence, progression and metastasis [12–15]. These compounds exhibit anti-proliferative, anti-inflammatory, anti-angiogenic, anti-oxidant and pro-apoptotic activities [12–15]. Major features of these agents are represented by low toxicity on normal cells of the host, and high tolerability over long term administration [12–15].

Using a novel highly sensitive mass spectrometry approach based on Surface-Activated Chemical Ionization (SACI) with an Electrospray Ionization (ESI) source and bioinformatics analyses (SANIST platform) for biomarker discovery, we had found that 3 molecules from the carnitine family were significantly decreased in serum sample from PCa patients, as compared to controls [16]. Based on these results, we hypothesized that carnitine might exert a potential protective role against prostate cancer. This hypothesis was further supported by evidence in the literature, demonstrating that carnitine supplementation results in limited tumour growth in many experimental models [17–21].

Acetyl-L-Carnitine (ALCAR), the acetylated derivative of carnitine, is involved in trans-mitochondrial membrane trafficking of acetyl units in catabolic and anabolic pathways. ALCAR is a master regulator for the generation of cellular energy and controls metabolic pathways [22]. Several studies have demonstrated the anti-inflammatory, anti-oxidant and free radical scavenging properties of ALCAR, as well as its stabilizing effects on mitochondrial membrane [22]. ALCAR has been shown to exert beneficial effects in disorders where the oxidative stress acts as a promoting factor [17, 20, 23–26], such as diabetes, Alzheimer’s [26, 27]. Considering the pleiotropic beneficial actions, excellent safety and tolerability profile, ALCAR has been employed in clinical settings related to neurological disorders [26, 28, 29].

We have recently demonstrated that ALCAR has angiopreventive activities on endothelial cells acting on the VEGF/VEGFR2 and CXCR4/CXCL12 axes [30]. ALCAR also blocked the activation of NF-κB and ICAM-1 and inhibited inflammatory angiogenesis in vivo*,* interfering with endothelial cell and macrophage recruitment [30]. Based on the extensively reported antioxidant and anti-inflammatory properties of ALCAR, we investigated the ability of ALCAR to interfere with key functional steps of prostate carcinogenesis and identified some molecular mediators involved. We explored the possibility of targeting PCa by limiting the production/release of pro-inflammatory/pro-angiogenic cytokines and chemokines by ALCAR in vitro and tumour cell growth in vivo.

To define which pro-inflammatory/pro-angiogenic cytokines and chemokines could be modulated by ALCAR in PCa, for perspective future clinical trials, we performed cytokine profile analysis and in vitro studies, using four PCa cell lines (PC-3, DU-145, LNCaP, 22Rv1) and one benign prostatic hyperplasia (BPH) cell line. We found that treatment of the selected PCa and BPH cell lines with ALCAR resulted in decreased production and release of pro-inflammatory/pro-angiogenic cytokines, such as TNF-α, CCL2, IL-6, CXCL12, CXCL8 and VEGF. Functional assays recapitulating the pro-tumour behaviour and progression, showed that ALCAR reduces cell growth and hinders PCa and BPH cell migration, and invasion, and limits MMP-9 production.

We also found that ALCAR interferes with the paracrine effects of PCa secreted products on endothelial cells. ALCAR downregulated the expression of cognate chemokine receptors on endothelial cells as well as capillary morphogenesis. Finally, oral administration of ALCAR, resulted in reduced tumour volume and weight of DU-145 and 22Rv1 in in vivo xenograft models.

Our results places ALCAR as a compound with chemopreventive and angiopreventive properties, acting on pathways involved in inflammation and angiogenesis. We propose to test ALCAR as a potential “repurposed drug” for cancer chemoprevention, similar to metformin, aspirin or betablockers [31–33].

## Methods

### Chemicals, cells and cell culture

Crystal violet staining solution and L-acetyl-Carnitine (ALCAR) were purchased by Sigma Aldrich. Prostate cancer (PCa) cell lines (PC-3, DU-145, LNCaP, 22Rv1) and benign prostate hyperplasia cell line (BPH) were purchased by American Type Culture Collection (ATCC) and cultured in RPMI, 10% Foetal Bovine Serum (FBS), 1% Glutamine, 1% PenStrept, at 5% CO_2_ and 37 °C. Human umbilical vein endothelial cells (HUVEC, Lonza) were cultured in endothelial cell basal medium (EBM™, Lonza) supplemented with endothelial cell growth medium (EGM™SingleQuots™, Lonza), 10% FBS, 2 mM L-glutamine, 100 U/mL penicillin and 100 μg/mL streptomycin. HUVECs were used between the 3–5 passages. ALCAR treatment was performed in serum-free RPMI, 1% Glutamine, 1% pen-strep. To obtain conditioned media (CM), cells were pre-treated for 24 h with ALCAR (1 or 10 mM) and collected. Residual cells, debris were discarded by centrifugation and CM concentrated with concentricon devices (Millipore, Temecula, CA) with a 3-kDa membrane pore cut-off, which eliminates the residual ALCAR.

### Cell viability assay

The viability of cells was determined by using crystal violet staining solution. 2 × 10^3^ cells (PC-3, DU-145, LNCaP, 22Rv1, BPH) were seeded into 96-well plates and treated with ALCAR (range: 50 μM, 100 μM, 200 μM, 500 μM, 1 mM, 5 mM or 10 mM) for 24, 48 and 72 h. After washing, cells were incubated with 50 μl of crystal violet staining solution, for 20 min at room temperature, gently washed with distilled water and let air-drying for at least 2 h at room temperature. Cell-retained crystal violet was dissolved in 100 μL of crystal violet elution buffer (50% of ethanol and 0.1% acetic acid). Cell viability was determined by absorbance, at 595 nm wavelength, with a microplate reader in a SpectraMax M2 (Molecular Devices, Sunnyvale CA)**.**

### Detection of apoptosis

PC-3, DU-145, LNCaP and BPH cells were treated with 1 or 10 mM ALCAR for 24 and 48 h. To exclude potential toxic effects of ALCAR on normal cells, peripheral blood mononuclear cells (PBMCs) from healthy donors were also treated with 1 or 10 mM ALCAR for 24 and 48 h. Induction of apoptosis was detected by Propidium Iodide (PI, 1 μg/mL) (Sigma Aldrich) and Annexin-V-APC (Immunotools) staining, followed by flow cytometry analysis, using a BD FACSCantoII flow cytometer. Flow data were analyzed with the FACSDiva 6.1.2 software (Becton Dickinson-BD) and the FlowLogic (Miltenyi Biotec) software.

### Detection of cell cycle

Unsynchronized PC-3, DU-145, LNCaP and BPH cells were treated with ALCAR (1 or 10 mM), for 24 h. Following treatments, cells were fixed in 70% ice cold ethanol. Nuclei were stained with 10 μg/ml DAPI and analysed using a FACSCantoII flow cytometer. Cell population distributions in G0/G1, S, G2/M and apoptotic phase of the cell cycle were analyzed with FACSDiva (BD Biosciences) and FlowLogic (Miltenyi Biotech) softwares.

### Adhesion assay

PC-3, DU-145, LNCaP and BPH cells were pre-treated with 1 or 10 mM ALCAR for 24 h. Following treatment, 3 × 10^3^ cells were seeded on 8-well chamber slides pre-coated with 2 μg/ml fibronectin (Sigma Aldrich) [30], for 45 min at 37 °C, 5% CO^2^. Following 90 min of incubation, cells were washed with PBS, fixed with 4% paraformaldehyde (PFA) and stained with DAPI 1 μg/mL (Sigma Aldrich). Cells within three random fields for each condition were counted using a Zeiss microscope in a double-blind manner.

### Migration and invasion assay

PC-3, DU-145, LNCaP and BPH cells were pre-treated with 1 or 10 mM ALCAR for 24 h. Following treatment, a modified Boyden Chamber, as described in [34, 35] was used to perform migration and invasion assays. 25 × 10^3^ cells were added in the upper chamber of the Boyden apparatus. Then, 8 μm pore-size polycarbonate filters, pre-coated with fibronectin (2 μg/mL; migration assay) or with matrigel (1 mg/ml; Becton Dickinson; invasion assay) were used as interface between the two compartments. Medium supplemented with 10% FBS was placed in the bottom chamber of the Boyden system, to induce migration and invasion. Following 6 h (migration) or 24 h (invasion) of incubation at 37 °C in 5% CO_2_, migrated/invaded cells were counted. Briefly, filters were removed, fixed in absolute ethanol, re-hydrated in distilled water and stained with DAPI (10 μg/ml; Sigma Aldrich). Migrated/invaded cells were counted in a double-blind manner in 5 consecutive fields, using a Zeiss Microscope associated with a Nikon camera.

### Flow cytometry for cytokine/chemokine detection and MMP-9 production

The effects of ALCAR on the modulation of selected cytokines/chemokines and MMP-9 production was analyzed by flow cytometry. PC-3, DU-145, LNCaP and BPH cells were treated with ALCAR (1 or 10 mM) for 24 h. Following treatment, 3 × 10^5^ cells/per FACS tube were stained for 30 min at 4 °C, for surface antigen detection of CXCR4 (Clone #12G5, Biolegend). For intracellular cytokine and MMP-9 detection, PC-3, DU-145, LNCaP, BPH, and TNFα (10 ng/mL) pre-activated HUVECs (3 × 10^5^ cells/per FACS tube) were fixed and permeabilized, using the CytoFix/Cytoperm kit (Becton Dickinson), and stained with the following PE-conjugated Mabs: anti-human VEGF (Clone #23410, R&D Systems), CXCL12/SDF-1 (Clone #79018, R&D Systems); CCL2/MCP-1 (Clone #REA248, Miltenyi Biotec), TNF-α (Clone #REA656, Miltenyi Biotec), CXCL8 (Clone #E8N1, Biolegend), IL-6 (Clone #MQ2-13A5, Miltenyi Biotec). For MMP-9 detection, fixed and permeabilized cells were stained with the anti-human MMP-9 primary antibody (Abcam), followed by staining with the PE-conjugated secondary antibody (R&D System). We also evaluated the ability of conditioned media (CM) from pre-treated PCa and BPH cells with ALCAR (1 or 10 mM), to interfere with the expression of CXCR4 (receptor for CXCL12), CXCR1, CXCR2 (receptors for CXCL8) and CCR2 (receptor for CCL2) on human umbilical vein endothelial cells (HUVEC; Lonza). CMs were obtained from PC-3, DU-145, LNCaP and BPH cells, pre-treated for 48 h, with ALCAR (1 or 10 mM), in serum-free RPMI medium. Following 24 h of exposure to CMs, 3 × 10^5^ HUVE cells/per FACS tube were stained for 30 min at 4 °C with the following PE- conjugated mabs: anti-human CXCR4, anti-human CXCR1 (REAA958), anti-human CXCR2 (REA208), anti-human CCR2 (REA264), all purchased by Miltenyi biotech. Fluorescence intensity for surface antigens and intracellular cytokines was detected by flow cytometry, on viable (SSC Vs FSC) gated cells, using a BD FACS Canto II analyser. Flow data were analysed with the FACSDiva 6.1.2 software (Becton Dickinson) and the FlowLogic (Miltenyi Biotec) software. FACS data from VEGF, CXCL-8 and Angiogenin, were validated by Bioplex [30], on CMs from PC-3, DU-145 and LNCaP cells, following 24 h of treatment with ALCAR 1 mM.

### Western blot analysis

ALCAR ability to induce apoptosis in PCa and BPH cells was confirmed by western blotting. Following 24 h of treatment with ALCAR (1 or 10 mM), cells were lysed in RIPA buffer, supplemented with protease and phosphatase inhibitor cocktails (Roche Diagnostics GmbH). Proteins (30 μg) were separated on the NupageNovex on 4–12% Bis-Tris Gel (Life Technologies) and transferred to a PVDF membrane Amersham Hybond (GE Healthcare Biosciences). Membranes were incubated overnight at 4 °C with Cleaved Caspase-3 (Asp175) (Cell Signalling Technology) and with peroxidase-linked anti-rabbit IgG or anti-mouse IgG secondary antibodies (GE Healthcare Life science) for 1 h at room temperature. Specific protein bands were detected with Pierce ECL Western Blotting Substrate (ThermoFisher Scientific). Protein expressions were normalized to beta-Actin (Abcam). Band intensity (revealed as optical density-OD) were detected by ImageJ software.

### Effects of conditioned media (CM) from PCa cell lines on endothelial cells morphogenesis

We investigated the ability of ALCAR to limit the induction of angiogenesis by DU-145 cells, via soluble factors. Collected CMs were used to assess their ability to induce capillary-like structures of HUVE cells on matrigel. 15 × 10^3^ HUVECs were seeded into a 96well plate, previously coated with 1 mg/mL of reduced growth factor Matrigel (Becton Dickinson). HUVECs received the collected CMs (50 μg total protein) from DU-145, in FBS-free EBM medium and were incubated for 6 h. Positive controls received 10% FBS EBM medium. The capillary network formation was determined using a Zeiss Microscope associated with a Nikon camera (Axio Observer A1, Zeiss) and quantified with ImageJ software (U.S. National Institute of Health, Bethesda, MD, USA), using the Angiogenesis Analyzer tool [30].

### Growth in in vivo xenografts models

The effects of ALCAR in inhibiting PCa tumor cell growth was assessed using an in vivo xenograft model. Nu/MRI nude mice (males, 5 weeks of age, from Charles River) were used. Animals were housed in a conventional animal facility with 12 h light/dark cycles and fed ad libitum. Four animals per group were subcutaneously injected into the right flank with 2.5 × 10^6^ DU-145 or 22Rv1 cells, in a total volume of 300 μL, containing 50% of serum free RMPI 1650, and 50% of 10 mg/mL reduced growth factor Matrigel (Corning) with or without ALCAR 10 mM. From day 0 animals received daily ALCAR 10 mM, corresponding to 20 mg/Kg b/w (Bulk Powders 100% in purity for human use; Essex,) in the drinking water. Tumour volume was measured with a calliper and determined using the formula (W^2^ × L)/2. At day 21 (22Rv1) and day 27 (DU-145) tumours were surgically excised, photographed, and weighted. Part of the tumours were used for histology. Formalin-fixed, paraffin-embedded serial tissue sections (5 μm of thickness) from control or ALCAR treated tumours (DU-145 and 22Rv1) were deparaffinised with xylene and rehydrated in graded ethanol, followed by haematoxylin and eosin staining. Vessel numbers were manually counted on single sections. All the procedures involving the animals and their care were according to the institutional guidelines, in compliance with national and international law and guidelines for the use of animals in biomedical research and housed in pathogen-free conditions. All the procedure applied were approved by the local animal experimentation ethics committee (ID# #06_16 Noonan) of the University of Insubria and by the Health Ministry (ID#225/2017-PR).

### Statistical analysis

The statistical significance between multiple data sets was determined by one-way ANOVA. Differences between tumour growth and weights within the experimental groups were determined by two-way ANOVA and t-test, respectively. All the analyses were performed using Graph-Pad PRISM 7 and 8. Data are showed as mean ± SEM.

## Results

### ALCAR reduces PCa cell viability and induces apoptosis

**Fig. 1:**
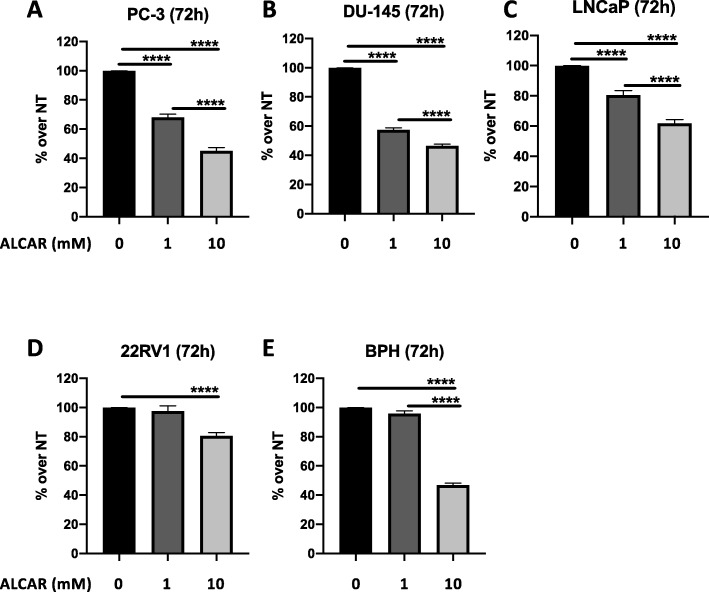
Effects of ALCAR 1 and 10 mM on PCa and BPH cell lines proliferation. PC-3 (**a**), DU-145 (**b**), LNCaP (**c**), 22Rv1 (**d**) and BPH (**e**) were treated with ALCAR 1 or 10 mM at 72 h. The proliferation rate was measured by Crystal Violet assay (O.D. 595 nm). Results are showed as normalize on control (not treated, NT), mean ± SEM, ANOVA, ****p < 0.0001

**Fig. 2:**
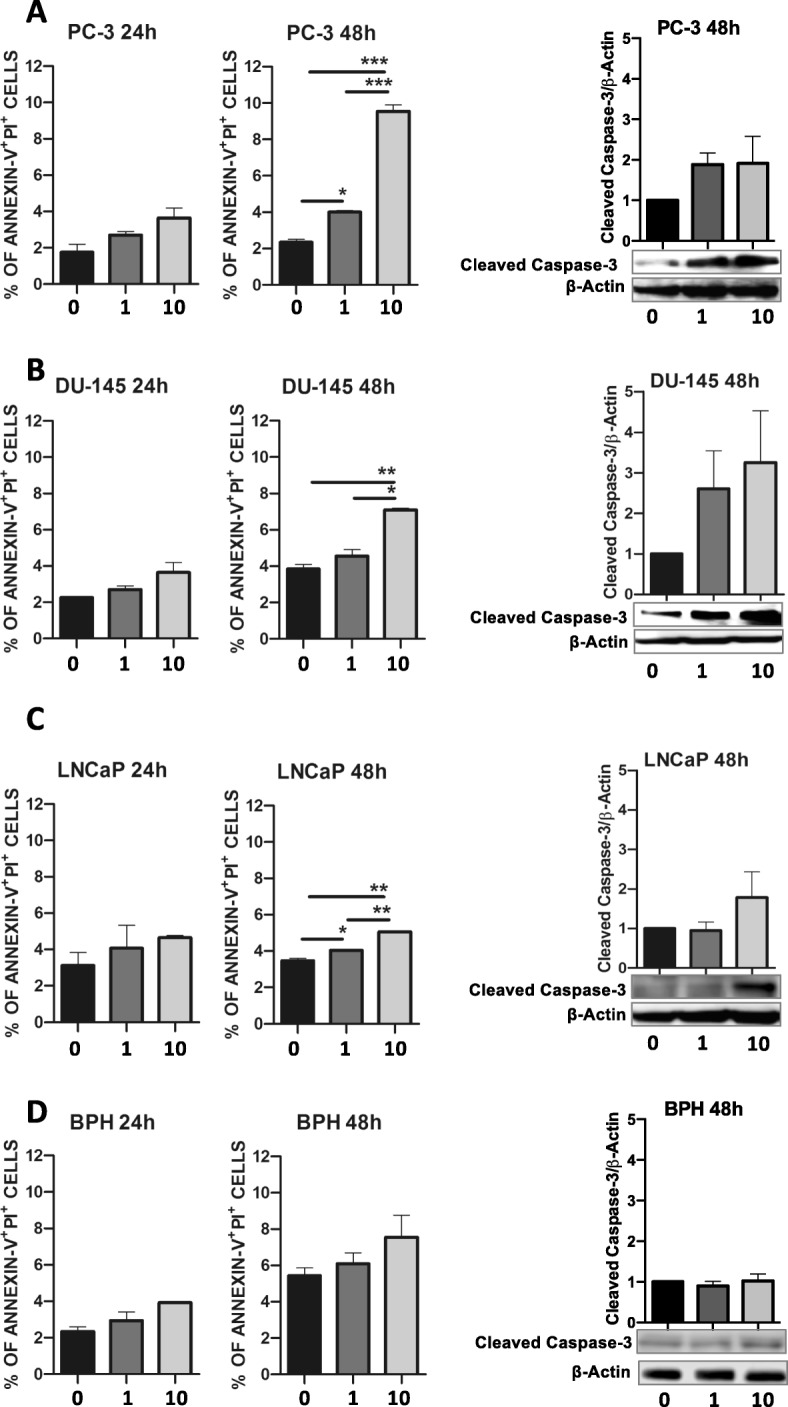
Effects of ALCAR in the induction of apoptosis in PCa and BPH cell lines. Induction of apoptosis was assessed, by flow cytometry, on (**a**) PC-3, (**b**) DU-145, (**c**) LNCaP and (**d**) BPH cells treated with ALCAR (1 or 10 mM) after 24 and 48 hours of treatment. Representative western blot images showing an up-regulation of Cleaved Caspase-3 (Asp175) in PCa cells treated with ALCAR 1 and 10 mM for 48 h. The graphs show quantification of Cleaved Caspase-3 (Asp175) normalised to β-Actin and to control (0). Results are shown as Mean ± SEM, ANOVA, *p < 0.05, **p < 0.01, *** *p* < 0.001

We tested the ability of ALCAR at different concentrations (50 μM, 100 μM 200 μM, 500 μM, 1 mM, 5 mM and 10 mM) to reduce cell growth in four PCa (PC-3, DU-145, LNCaP, 22Rv1) and BPH cell lines, by using crystal violet assay (data not shown). We identified ALCAR 1 and 10 mM as effective concentrations in limiting PCa cell proliferation. Treatments became statistically significant at 72 h of treatment (Fig. 1a-d). For BPH cells, we found that ALCAR was effective only at 10 mM (Fig. 1e). The selected ALCAR concentrations are in line with our previously results on endothelial cells, and other preclinical and clinical studies [17, 20, 24, 30, 36]. The effect of ALCAR on cell growth was in part due to induction of apoptosis, as determined by flow cytometry. ALCAR pro-apoptotic effect became statistically significant following 48 h of treatment for the PCa cell lines (Fig. 2a-d, Additional file 1: Figure S1 a-d). Caspase activation is one of the major mediators of the apoptotic response. Biochemical analysis showed that PCa and BPH cells treated with ALCAR increased the amount of cleaved caspase-3, (effector caspase of apoptosis) (Fig. 1a-d). In order to verify the safety/low toxicity of the employed concentrations, we examined the possible apoptotic effects of ALCAR on peripheral blood mononuclear cells (PBMCs) from healthy donors. We found that most normal cells remain viable (Additional file 1: Figure S1e). Finally, we observed no effects of ALCAR in inducing cell cycle arrest, both in the PCa and BPH cell lines (Additional file 1: Figure S2 a-d).

### ALCAR impairs PCa and BPH cells adhesion, migration and invasion in vitro

**Fig. 3:**
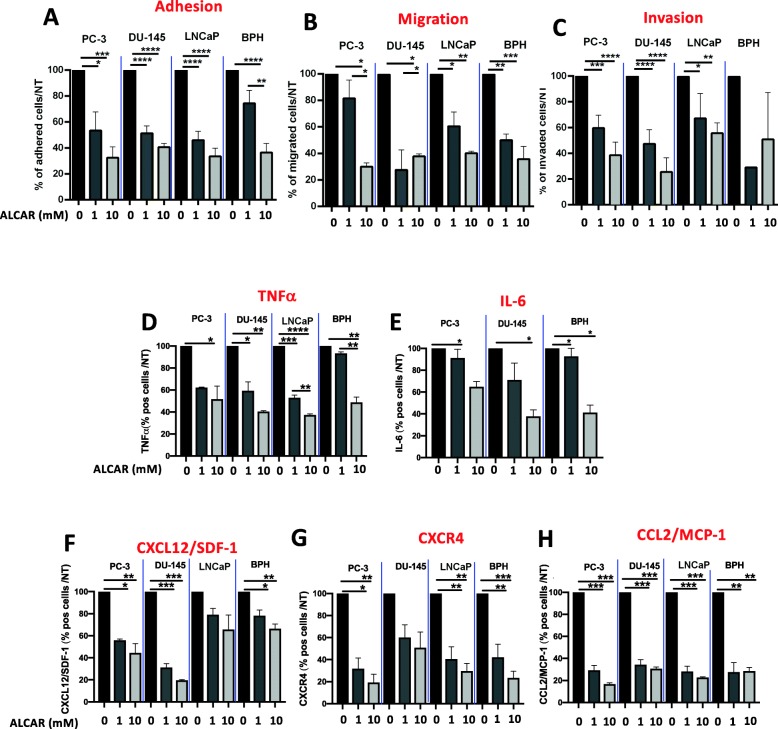
Effects of ALCAR on PCa and BPH cell lines functional assays and pro-inflammatory cytokine/chemokine release. To determine the effects of ALCAR on PCa and BPH cell lines adhesion, migration and invasion. PCa (PC-3, DU-145, LNCaP) and BPH cell lines were pre-treated for 24 h with ALCAR 1 and 10 mM and tested for the ability to of ALCAR prevent cell (**a**) adhesion on fibronectin (**b**) migration on fibronectin and (**c**) invasion in matrigel, using the Boyden chamber assay. ALCAR 1 and 10 mM were able to significantly inhibit cell adhesion, migration and invasion in the PCa and BPH cell line. PCa (PC-3, DU-145, LNCaP) and BPH were treated with ALCAR 1 and 10 mM for 24 h and analysed for pro-inflammatory cytokine production by flow cytometry. Histogram for pro-inflammatory and pro-migratory/invasive cytokines showing that ALCAR 1 and 10 mM reduced the expression of (**d**-**h**) TNF-α, IL-6, CXCL12-CXCR4 and CCL2 as fold-change over non-treated cells (NT). Results are showed as normalised over non-treated cells (NT), as mean ± SEM, ANOVA, *p < 0.05, **p < 0.01, ***p < 0.001, ****p < 0.0001

Tumour cell adhesion to extracellular matrix proteins such as collagen, fibronectin or laminin is crucial not only to facilitate cell proliferation but also to provide a support for migration and metastasis. We found that 24 h of pre-treatment with ALCAR (1 and 10 mM), resulted in inhibited adhesion of PC-3, DU-145, LNCaP and BPH cells on a fibronectin layer, in a concentration-dependent manner (Fig. 3a). Furthermore, PC-3, DU-145, LNCaP and BPH cells pre-treated for 24 h with ALCAR (1 or 10 mM), exhibited reduced migration (Fig. 3b) and invasion (Fig. 3c).

### ALCAR downregulates pro-inflammatory cytokine/chemokine involved in PCa and BPH cells

**Fig. 4:**
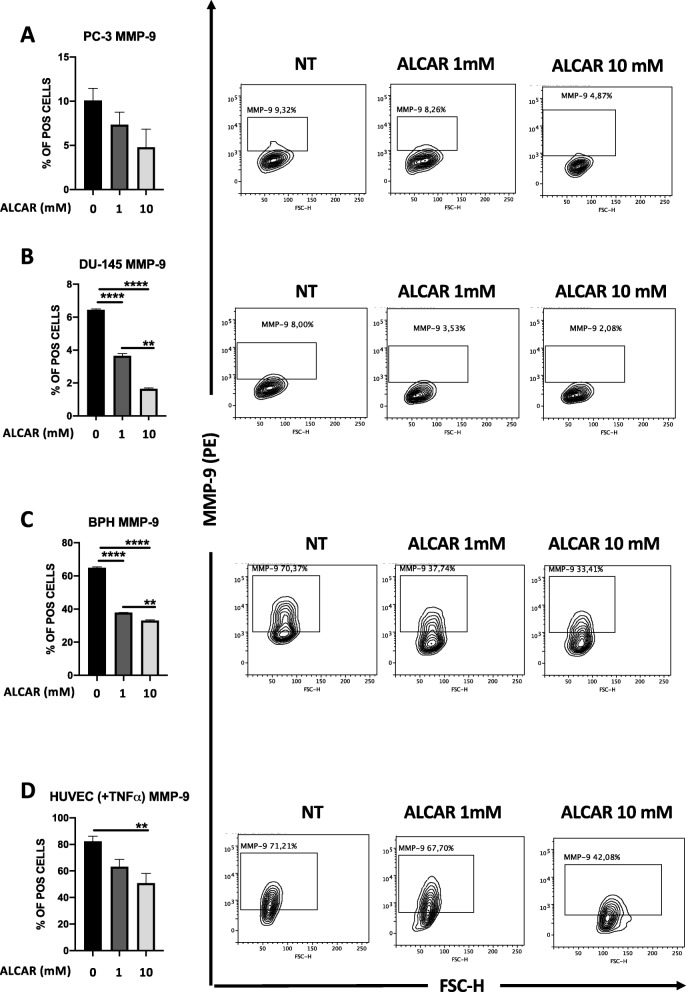
Effects of ALCAR on MMP-9 production in PCa, BPH cell lines and TNFα pre-activated HUVEC cells. PCa (PC-3, DU-145), BPH cell lines and TNFα pre-activated HUVEC cell were pre-treated for 24 h with ALCAR 1 and 10 mM and tested for the ability to produce MMP-9 by flow cytometry. Bar histograms and representative contour plots showing that ALCAR was able to limit MMP-9 production in PC-3, DU-145, BPH cell lines, and TNFα pre-activated HUVEC cell (**a**, **b**, **c** and **d**). Blockade of MMP-9 production by ALCAR was statistically significant in DU-145 and BPH cell lines. TNFα pre-activated HUVEC cell had a significant reduction in MMP-9 production upon ALCAR treatment. Results are showed as normalised over non treated cells (0), as mean ± SEM, ANOVA, ***p < 0.001, ****p < 0.0001

Evidence from the clinic indicates that prostate cancer progression correlates with elevated inflammation [3, 8–11, 37, 38]. We investigated whether ALCAR is able to interfere in vitro with the expression of TNF-α, together with other pro-inflammatory cytokines/chemokines such as IL-6, CCL2, as well as CXCL12 and its receptor CXCR4 on PCa (PC-3, DU-145, LNCaP) and BPH cells. PCa and BPH cells showed significant decrease in TNF-α levels as well as in IL-6, and CCL2 (Fig. 3d-h) following 1 and 10 mM ALCAR treatment. CXCL12 and its receptor CXCR4 are known as key regulators of highly migratory/invasive phenotype of prostate cancer and their expression is associated with metastatic disease and poor survival [39–41]. Additionally, ALCAR was able to significantly down-regulate the pro-migratory pathway CXCL12/CXCR4 that drives metastatic features in PCa (Fig. 3f-g, Additional file 1: Figure S3). Functional assays showed that ALCAR inhibits PCa and BPH cell migration and/or invasion through matrigel (Fig. 3c-d). CCL2 (Fig. 3h) which is also involved in PCa invasion and pro-metastatic features [42] was significantly inhibited in all PCa and BPH cell lines. CXCL12/CXCR4 signalling activates MMP-9 expression in prostate cancer cells [43]. Consistently, we found that reduction of PC-3, DU-145, LNCaP, BPH invasive capabilities correlates with decreased production of MMP-9, as revealed by FACS analysis (Fig. 4a-c). MMP-9 reduced expression was observed also in TNFα pre-activated HUVEC cells (Fig. 4d).

### ALCAR limits PCa induced pro-angiogenic features and regulates CXCL8-CXCR1/2, CCL2-CCR2 and CXCL12-CXCR4 axis in endothelial cells

**Fig. 5:**
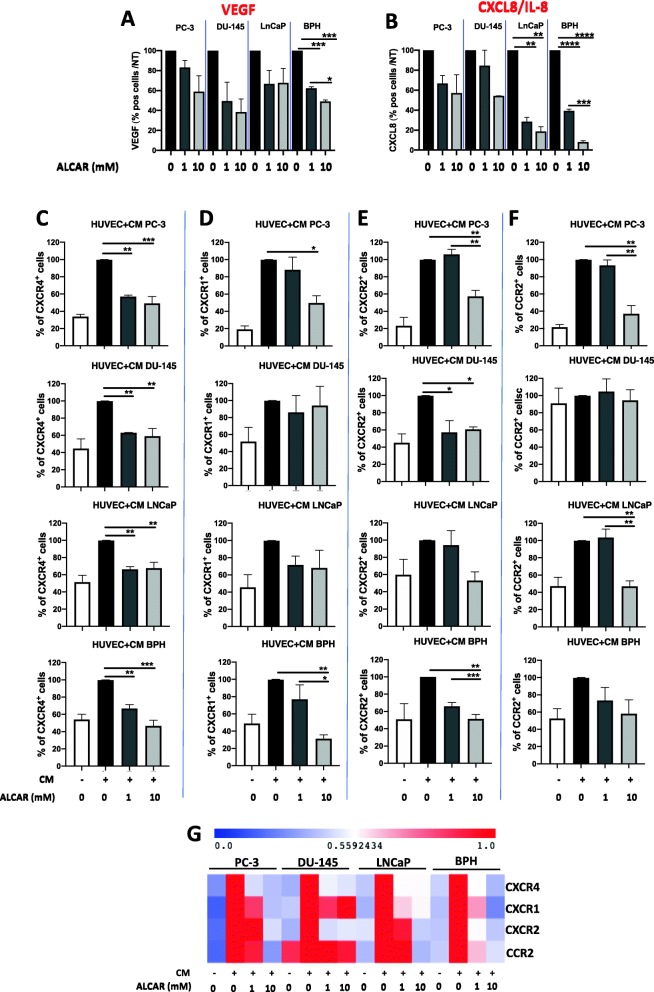
Effects of ALCAR on pro-angiogenic factors and endothelial cell chemokine receptors. PCa (PC-3, DU-145, LNCaP) and BPH were treated with ALCAR 1 and 10 mM for 24 h and analysed for pro-angiogenic cytokine production by flow cytometry. ALCAR 1 and 10 mM reduced the expression of (**a**) VEGF and (**b**) CXCL8. Conditioned media (CM) from PCa (PC-3, DU-145, LNCaP) and BPH cell lines were collected following 24 h of treatment with ALCAR 1 and 10 mM. Human Umbilical Vein Endothelial Cells (HUVEC) were treated with 50 μg/ml of CM from PCa (PC-3, DU-145, LNCaP) and BPH cell line and assessed by flow cytometry for the expression the chemokine receptors (**c**) CXCR4, (**d**) CXCR1, (**e**) CXCR2 (**f**) CCR2. (**g**) Representative heatmap for the investigated chemokine receptors as fold-change over non treated cells (0). Results are showed as normalised over non treated cells (0), as mean ± SEM, ANOVA, *p < 0.05, **p < 0.01, ***p < 0.001

The vascular endothelial growth factor (VEGF) and CXCL8, a CXC inflammatory chemokine, have been demonstrated to induce angiogenesis and promote progression of PCa [44, 45]. We investigated whether ALCAR was effective in limiting the release of pro-angiogenic factors in PC-3, DU-145, LNCaP and BPH cell lines. FACS analysis showed decreased trend in the production of VEGF, CXCL8 by three PCa and the BPH cell lines exposed to ALCAR 1 and 10 mM, following 24 h of treatment (Fig. 5a-b). We previously reported that ALCAR inhibits inflammatory angiogenesis in vitro and in vivo. Since chemokine receptors are key downstream effectors of angiogenesis induction, we evaluated the regulation of CXCR4, CXCR1, CXCR2, CCR2 expression on endothelial cells, incubated with conditioned medium from ALCAR treated PCa and BPH cell lines. We found a significant downregulation of CXCR4, CXCR1, CXCR2, CCR2 receptors on HUVEC cells exposed to cell products/CMs from all the PCa and BPH cell lines (Fig. 5c-g).

### ALCAR functionally inhibits angiogenesis in vitro

**Fig. 6:**
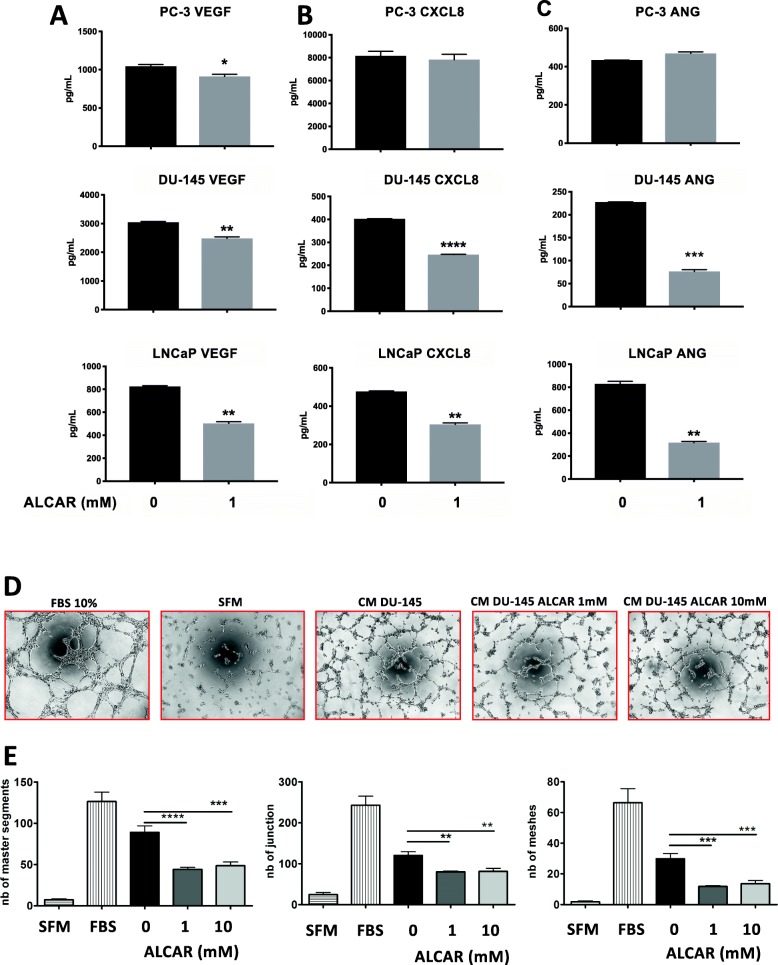
Indirect effects of ALCAR on capillary-like structure formation of HUVECs. Secretome profiling on PCa cell lines in response to ALCAR treatment by BIOPLEX analyses showing ALCAR ability to limit (**a**) VEGF, (**b**) CXCL8 and (**c**) Angiogenin (ANG) release on PCa cell lines (PC-3, DU-145, LNCaP) following 24 h of treatments with ALCAR 1 mM. (**d**, **e**) Morphogenesis assay on HUVECs pre-treated with 50 μg/ml of CM collected from DU-145 following 24 h of treatment with ALCAR 1 and 10 mM. (**d**) Representative images of tubular structures photographed at 5× magnification and (**e**) quantified by the Angiogenesis analyser ImageJ tool kit. HUVEC exposed to CM from DU-145 treated with ALCAR 1 and 10 mM exhibit a reduced ability to form capillary-like structures on Matrigel. SFM: cells cultured in serum-free EBM-2 medium; FBS+: cell treated with complete EGM-2 medium. Data are showed as Mean ± SEM. **p < 0.01, ***p < 0.001, ****p < 0.0001

We have previously shown that ALCAR inhibits endothelial cells by regulating angiogenesis in hypoxic and inflammatory conditions in vitro and in vivo [30]. Since we found a down regulation of pro-angiogenic factors VEGF, CXCL8 in PCa cells and chemokine receptors (CXCR1/2, CXCR4, CCR2) on endothelial cells, we assessed whether ALCAR 1 or 10 mM, would affect PCa cellc pro-angiogenic products release and consequently interfere with the ability of endothelial cells to functionally induce cell morphogenesis. We examined if ALCAR interferes with the VEGF and CXCL8 production in the conditioned media (CM), by evaluating the release of the same cytokines by BIOPLEX assay. We detected a statistically significant reduction in VEGF in three PCa cell lines (PC-3, DU-145, LNCaP) whereas CXCL8 release was significantly downregulated mostly in DU-145 and LNCaP cells exposed to ALCAR (Fig. 6a-b). We also evaluated by a multiplex Immunoassay (Bioplex) the release of the angiogenic factor angiogenin (ANG) after ALCAR treatment. Angiogenin release was significantly reduced in DU-145 and LNCaP cell lines following treatment (Fig. 6c).

Finally, we verified whether CM from DU-145 (as a representative PCa cell line) that were previously treated with ALCAR 1 and 10 mM, were able to induce network formation on HUVECs. We observed that CM from DU145 induced capillary-like network formation on a matrigel layer and that CM from PCa cells preteated with ALCAR had significantly reduced this capability; as showed by the quantification of: the number and total length of master segments and the number, total meshes area and number of junctions (Fig. 6d). These results confirm that PCa treated by ALCAR are less pro-angiogenic in vitro by inducing less HUVE capillary-like morphogenesis.

### ALCAR inhibits PCa cell growth in vivo

**Fig. 7:**
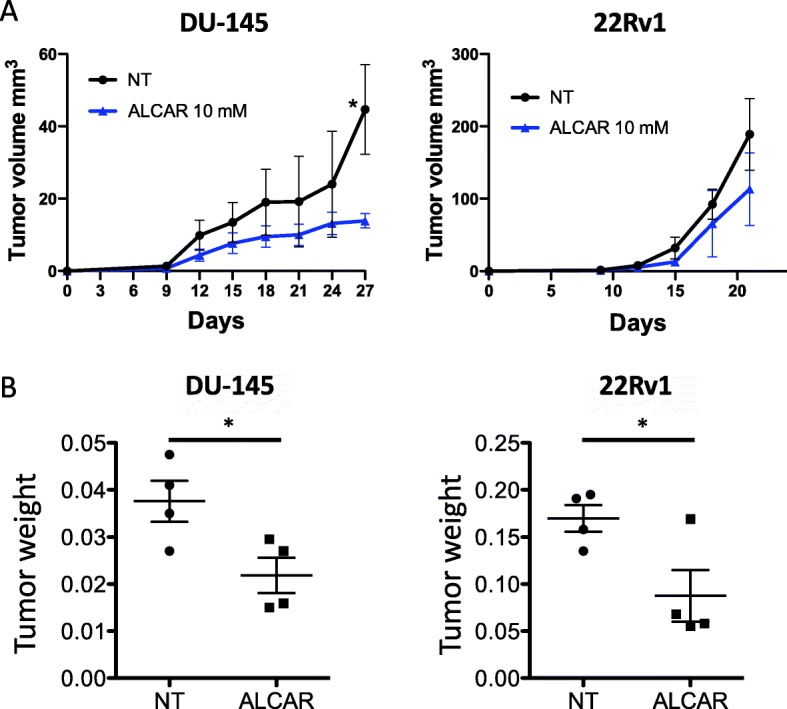
Effects of ALCAR on PCa cell growth in vivo. The effects of ALCAR (10 mM) was evaluated in vivo, using xenograft murine models in Nu/MRI nude mice. Four animals per experimental group were used. ALCAR reduced tumour volume (**a**) (mean ± SEM, two-way ANOVA, *p < 0.05) and weight (**b**) (mean ± SEM, t-test, *p < 0.05), in DU-145 and 22Rv1 xenografts

We investigated whether ALCAR was effective in inhibiting tumour growth of PCa in vivo. We employed two different PCa cell lines (DU-145 and 22Rv1), that were xenografted in Nu/MRI nude mice by subcutaneous injection. Daily oral administration of 10 mM ALCAR in drinking water resulted in reduction of tumour volume (Fig. 7a) and statistically significant weight (Fig. 7b) for both DU-145 and 22Rv1 xenografted mice. No differences in food and water intakes were observed in the two experimental groups, during the entire term of the study (data not shown). We did not observe significant alteration in the body weight of the animals treated with ALCAR, as compared with the untreated groups. The histological analysis showed that DU-145 xenografts, exposed to ALCAR, had less cellularity as compared to untreated ones (Additional file 1: Figure S4A). In ALCAR-treated xenografts, we observed a trend toward a reduced microvascular density, as revealed by histology (Additional file 1: Figure S4).

## Discussion

Inflammation and angiogenesis are widely recognized as critical orchestrators in tumour insurgence and progression [46]. Inflammation is involved in PCa development and progression [37]. The inflammatory microenvironment contributes to prostate cancer initiation, promotion, and progression via a number of mechanisms, including increased levels of specific cytokines/chemokines [38]. Because of the high prevalence, lifetime risk, and relatively slow rate of progression, prostate cancer is an ideal candidate for chemoprevention and interception approaches.

Acetyl-L-carnitine (ALCAR), the acetyl ester of L-Carnitine, is endogenously synthesized in human or obtained from dietary sources with a higher bioavailability compared to L-carnitine. ALCAR plays an important role in fat metabolism and in the normal function of energy metabolism in most tissues. ALCAR has been shown to exert several potential beneficial effects on human health, including anti-inflammation, anti-oxidation, and immunomodulation effects [25, 47–49].

Carnitine has been reported as highly efficient in the downregulation of pro-inflammatory cytokines TNF-α and IL-6 in murine models of cancer cachexia [24, 36], liver fibrosis [23] and in blocking TNF-α-induced insulin resistance in skeletal muscle cells [48].

**Fig. 8:**
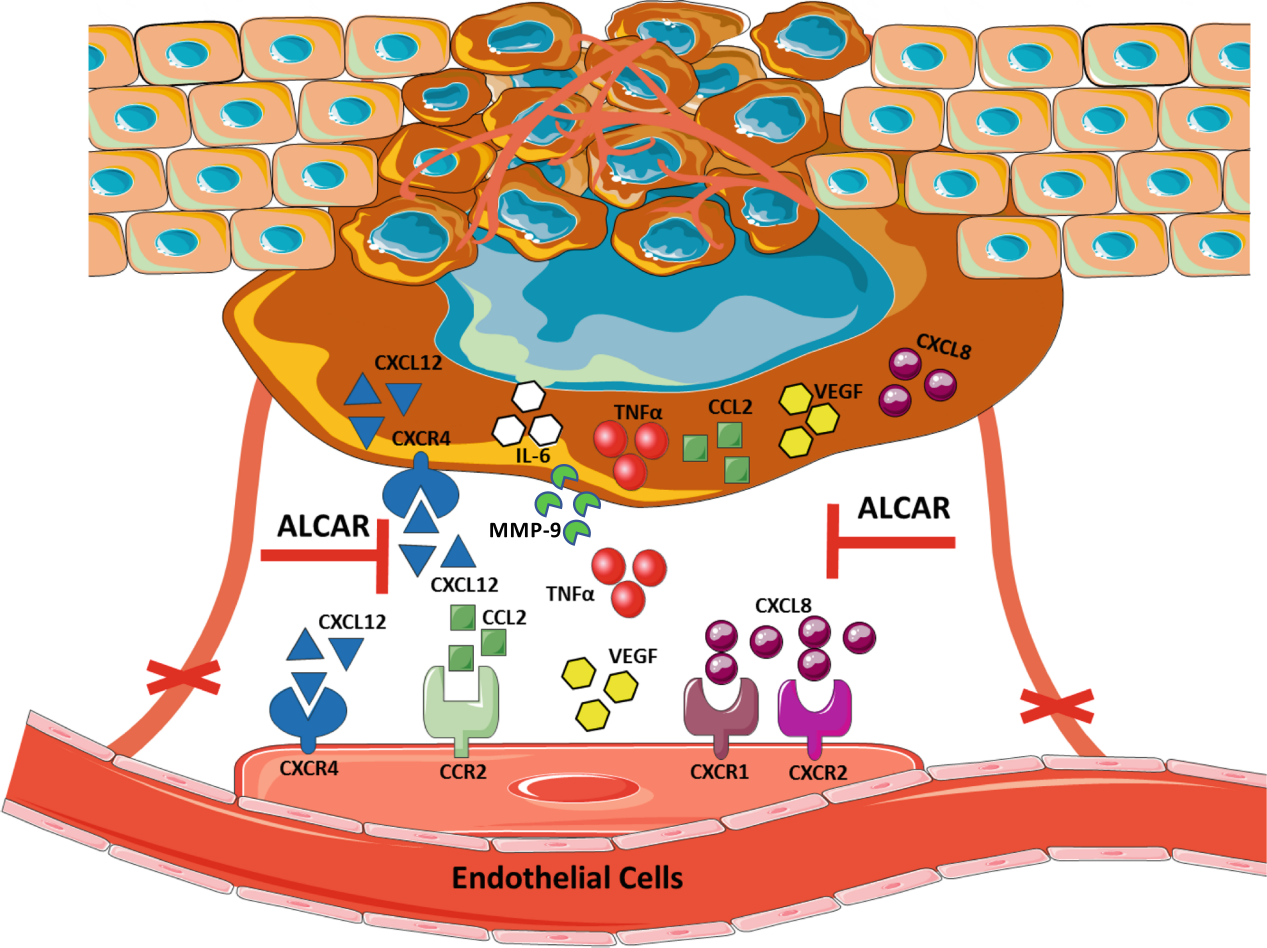
Schematic presentation of the anti-inflammatory and anti-angiogenic properties of ALCAR on PCa and BPH cells. Cytokines and chemokines (TNF-α, CCL2, IL-6, CXCL12, CXCL8 and VEGF) regulated in PCa cells and chemokine receptors (CXCR4, CXCR1, CXCR2, CCR2) in HUVEC cells targeted by ALCAR**.** Blocks indicate inhibited targets by ALCAR treatment observed in our experimental in vitro models

Given the high levels of pro-inflammatory related cytokines and relationship between prostate cancer and elevated inflammation, we investigated whether ALCAR can hinder the inflammatory and pro-angiogenic cytokine/chemokine levels. This is consistent with previous evidence suggesting that ALCAR alleviates inflammation and induces improvement in a large array of diseases [25, 47–49]. Here we show that ALCAR suppresses the release of crucial pro-inflammatory and pro-angiogenic factors. We analysed the changes in TNF-α, CCL2, IL-6, CXCL12, CXCL8 and VEGF levels and we found a reduced expression in response to ALCAR treatment of PCa and BPH cell lines (Fig. 8).

Chemokine (C-X-C motif) receptor 4 (CXCR4) expression increases during progression of PCa and is associated with metastatic disease and poor survival [50, 51] CXCR4 selectively binds to stromal cell-derived factor 1 (SDF-1)/ CXCL12, also overexpressed in PC metastatic tissue compared to normal tissues [52]. The CXCL12/CXCR4 axis has been shown to play an important role in PC cell proliferation, migration and invasion [53–55]. We found that ALCAR was able to limit the production of CXCR4 and CXCL12 in PCa (PC-3, LNCaP, DU145) and BPH cell lines, suggesting a potential role in limiting the induction of a migratory phenotype (Fig. 8). Chemoattractive mechanisms involving migration of PCa cells leads to the activation of multiple signalling pathways and subsequent secretion of MMP-9 into the local environment. ALCAR inhibits MMP9 which could be involved in invasive features of PCa and BPH cell lines. These data were supported by functional studies, and we found that ALCAR can inhibit hallmarks of tumour progression in vitro, by limiting PC-3, LNCaP, DU145 and BPH cell adhesion, migration and invasion. These activities of ALCAR were observed not only in androgen-dependent (LNCaP cells) and androgen-independent metastatic PCa cells (PC-3 and DU145), but also in BPH cell lines, which represent a pre-neoplastic stage.

We recently reported that ALCAR was able to act on endothelial cells interfering with the VEGF/VEGFR2 and CXCR4/CXCL12 axes and reducing angiogenesis in vitro. We also found that ALCAR inhibits angiogenesis in vivo, by reducing endothelial cells and macrophage recruitment in the matrigel plugs [30] in mice. It is reported that angiogenesis and its major regulator VEGF are controlled by multiple transcription factors in the tumor microenvironment, including androgen receptors [56] and estrogen receptors [5, 57]. Androgen regulation is a very important point, but since the major prostate cancer cell lines used in the present study were not responsive to hormone levels, we did not assess changes in angiogenesis due to androgen administration. The role of androgen receptor in the regulation of angiogenetic growth factors in prostate cancer were not addressed as our major focus in this study. Since we found that VEGF baseline production was high in PC-3, DU-145 and LNCaP even in absence of dihydrotestosterone, we hypothesise that VEGF and angiogenesis are regulated by ALCAR via other mechanisms which might be independent from androgen receptors. Future investigations also by other groups could address also this question.

Angiogenesis induction provides the basis for fostering tumour growth and metastasis, and the related cytokine/chemokine milieu acts as an orchestrator for this process. The function of CXCL8 relies on its interaction with specific cell surface G protein-coupled receptors (GPCR), CXCR1 and CXCR2 triggering signalling pathways that mediate angiogenesis, and metastasis in PCa [58, 59]. CCL2 interaction with its cognate receptor CCR2 promotes PCa tumour growth by sustaining macrophage infiltration and angiogenesis [60–62]. Consistent with what we reported while studying endothelial cells [30], we found that ALCAR downregulates in PCa cells the release of pro-angiogenic factors involved in inflammation and endothelial recruitment and activation such as VEGF, CXCL8, CCL2 and CXCL12.

We also assessed the modulation of expression of CXCR1 and CXCR2 (CXCL8 cognate receptors) on endothelial cells exposed to conditioned media collected from PCa and BPH cell lines previously treated with ALCAR 1 and 10 mM. We observed downregulation of CXCR1 and CXCR2 on HUVEC cells treated with conditioned media from PCa and BPH cells pre-treated to ALCAR. Given the ability of ALCAR to hinder CCL2 production in PCa and BPH cell lines, we also investigated effects of PCa conditioned media with or without ALCAR on CCR2 expression by endothelial cells, to assess paracrine mechanisms. We observed lower expression of CCR2 on HUVEC treated with PCa supernatant (PC-3, LNCaP) exposed 24 h to ALCAR.

Our results shed light on the ability of ALCAR to target cytokine/chemokine axis aberrantly activated in PCa cells and orchestrating PCA progression via inflammation and angiogenesis. ALCAR also effects the BPH cell line, further supporting its chemoprevention action. These promising results place ALCAR as an attractive supplement to be explored for prevention approaches. Translation of our results in vivo, showed the ability of ALCAR to reduce tumour volume and weight in two different xenograft murine models of PCa, Further studies and trials are necessary to assess the use of ALCAR as “repurposed” agent for chemo/angiopreventive agent for a direct transfer to the clinic.

## Conclusions

Taken together, our study suggests that ALCAR, endowed with anti-inflammatory and anti-angiogenic properties, interferes with biological activities representing hallmarks of cancer, and with production of chemokine /cytokine in pro-angiogenic axis, involved in PCa progression. We suggest that ALCAR could be employed as a potential chemopreventive supplement for prostate cancer, in a view of drug repurposing strategies. Clinical studies on effect of ALCAR in PCa or pre-neoplastic situations are warranted to support ALCAR as an interception agent in cancer.

## Supplementary information

**Additional file 1: Figure S1.** Effects of ALCAR in the induction of apoptosis inPCa and BPH cell lines. Graphs show the effect of ALCAR (1 and 10 mM, 24 and 48 h) in the induction of apoptosis in PC-3 (A), DU-145 (B), LNCaP (C), BPH (D) cell lines and peripheral blood mononuclear cells (PBMCs) (E) from heathy controls. Stacked graphs refer to different cell state: viable (AnnexinV^−^PI^−^ cells), early apoptotic (AnnexinV^+^PI^−^ cells), late apoptotic (AnnexinV^+^PI^+^ cells), apoptotic (AnnexinV^−^PI^+^ cells). **Figure S2.** Effects of ALCAR in the induction of cell cycle arrest in PCa and BPH cell lines. Graphs show the effect of ALCAR (1 and 10 mM, 24 and 48 h) on cell cycle in PC-3 (A), DU-145 (B), LNCaP (C), BPH (D) cell lines. Stacked graphs refer to different phase of cell cycle: S phase, Apoptosis, G2/M, G0/G1. No effects on cell cycle was observed. **Figure S3.** Cytokine/chemokine profiling on PCa and BPH cell lines in response to ALCAR treatment. Representative heatmap for cytokine modulation (CCL2, IL-6, TNF-α, CXCL12, CXCR4, VEGF and CXCL8), as fold-change over non treated cells (NT). **Figure S4.** Histological analysis of ALCAR treated DU-145 and 22Rv1 xenografts. Haematoxylin/eosin staining (10X magnification**)** for sections (5 μm) of excised tumours derived from DU-145 and 22Rv1 showing a trend of reduced microvascular density in ALCAR-treated animals (A-F). Arrows indicate vessels.

## Data Availability

N/A

## Abbreviations

ALCAR: Acetyl-L-carnitine
ANG: Angiogenin
BPH: Benign prostatic hyperplasia
CCL2: C-C Motif Chemokine Ligand 2
CCR2: C-C chemokine receptor type 2
CM: Conditioned media
CXCL12: C-X-C Motif Chemokine Ligand 12
CXCL8: C-X-C Motif Chemokine Ligand 12
CXCR1: C-X-C chemokine receptor type 1
CXCR2: C-X-C chemokine receptor type 2
CXCR4: C-X-C chemokine receptor type 4
DAPI: 4′,6-diamidino-2-phenylindole
EBM: Endothelial basal medium
EGM: Endothelial growth medium
FACS: Fluorescence activated cell sorting
FBS: Foetal bovine serum
FC: Flow cytometry
FSC: Forward-scattered light
ICAM-1: Intercellular adhesion molecule 1
IL-6: Interleukin-6
MCP-1: Monocyte chemoattractant protein 1
NFκB: Nuclear factor kappa-light-chain-enhancer of activated B cells
PBMCs: Peripheral blood mononuclear cells
PCa: Prostate cancer
SDF-1: Stem-derived facrior-1
SSC: Side-scattered light
VEGF: Vascular endothelial growth factor
VEGFR: Vascular endothelial growth factor receptor
