# Supplementary figures and images for: Acetyl-L-Carnitine downregulates invasion (CXCR4/CXCL12, MMP-9) and angiogenesis (VEGF, CXCL8) pathways in prostate cancer cells: rationale for prevention and interception strategies
Source: J Exp Clin Cancer Res. 2019 Nov 12;38:464. doi: 10.1186/s13046-019-1461-z (PMC6852951; doi:10.1186/s13046-019-1461-z)

Suppl. Figure 1

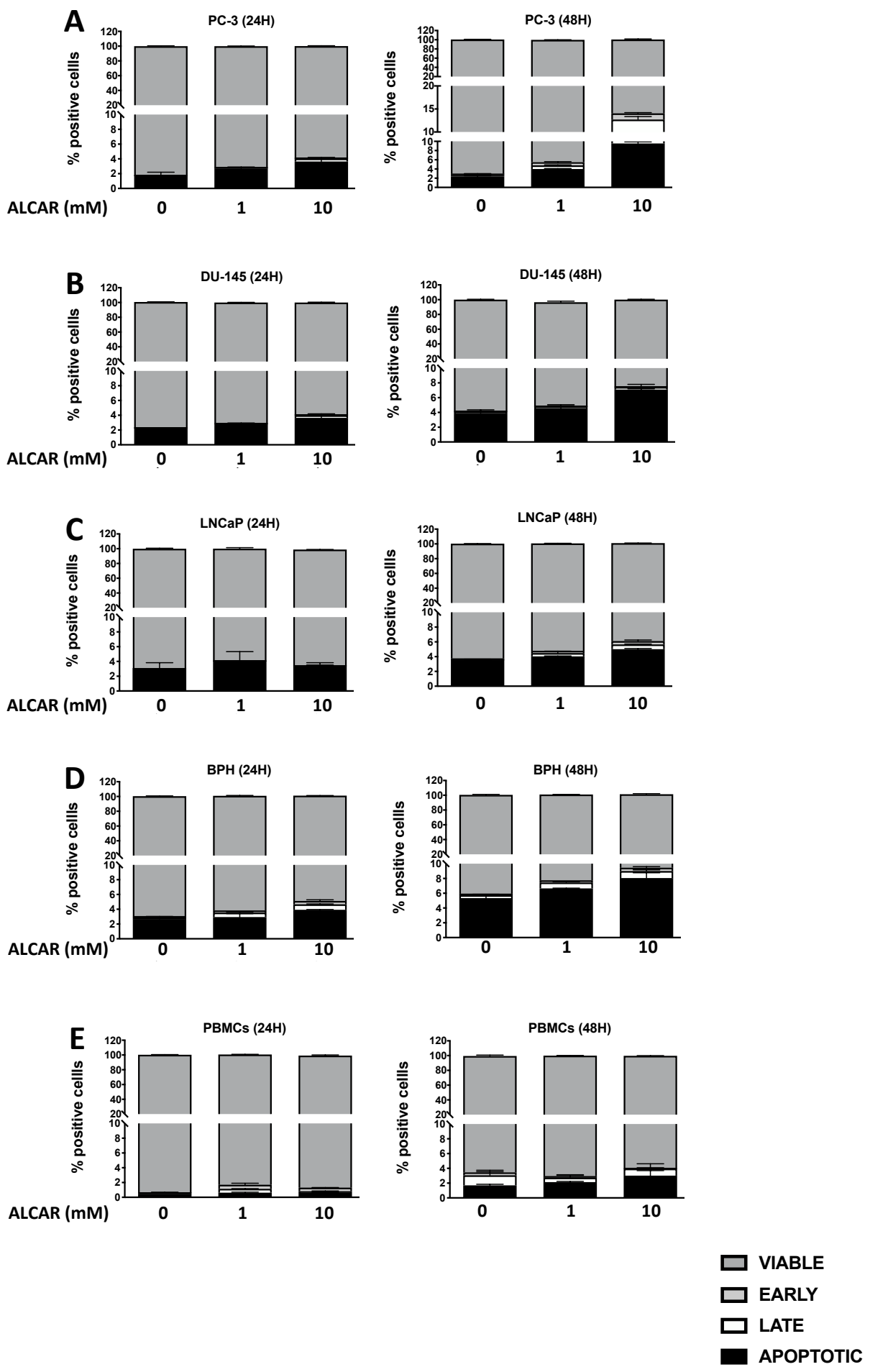

Suppl. Figure 2

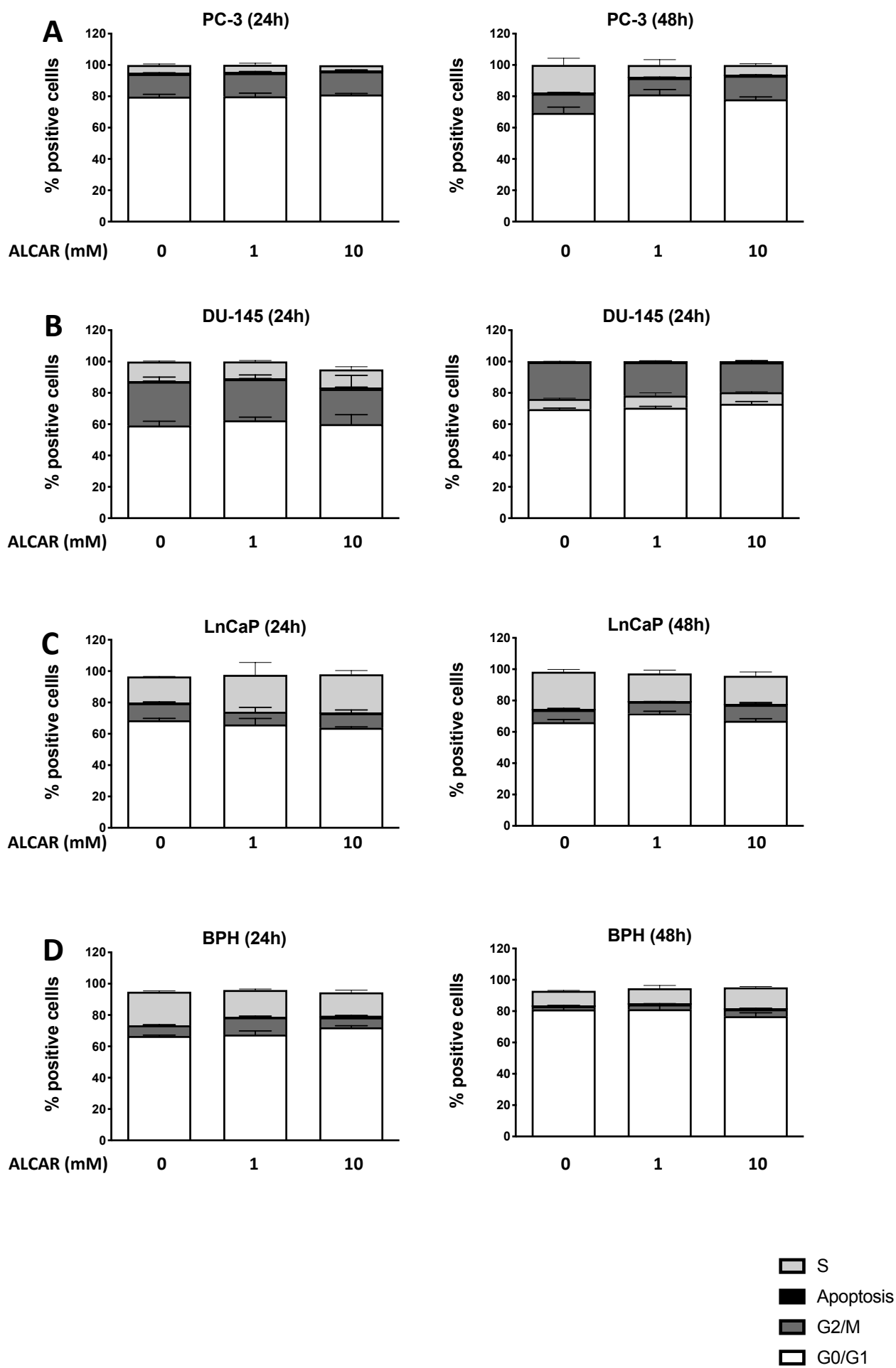

Suppl. Figure 3

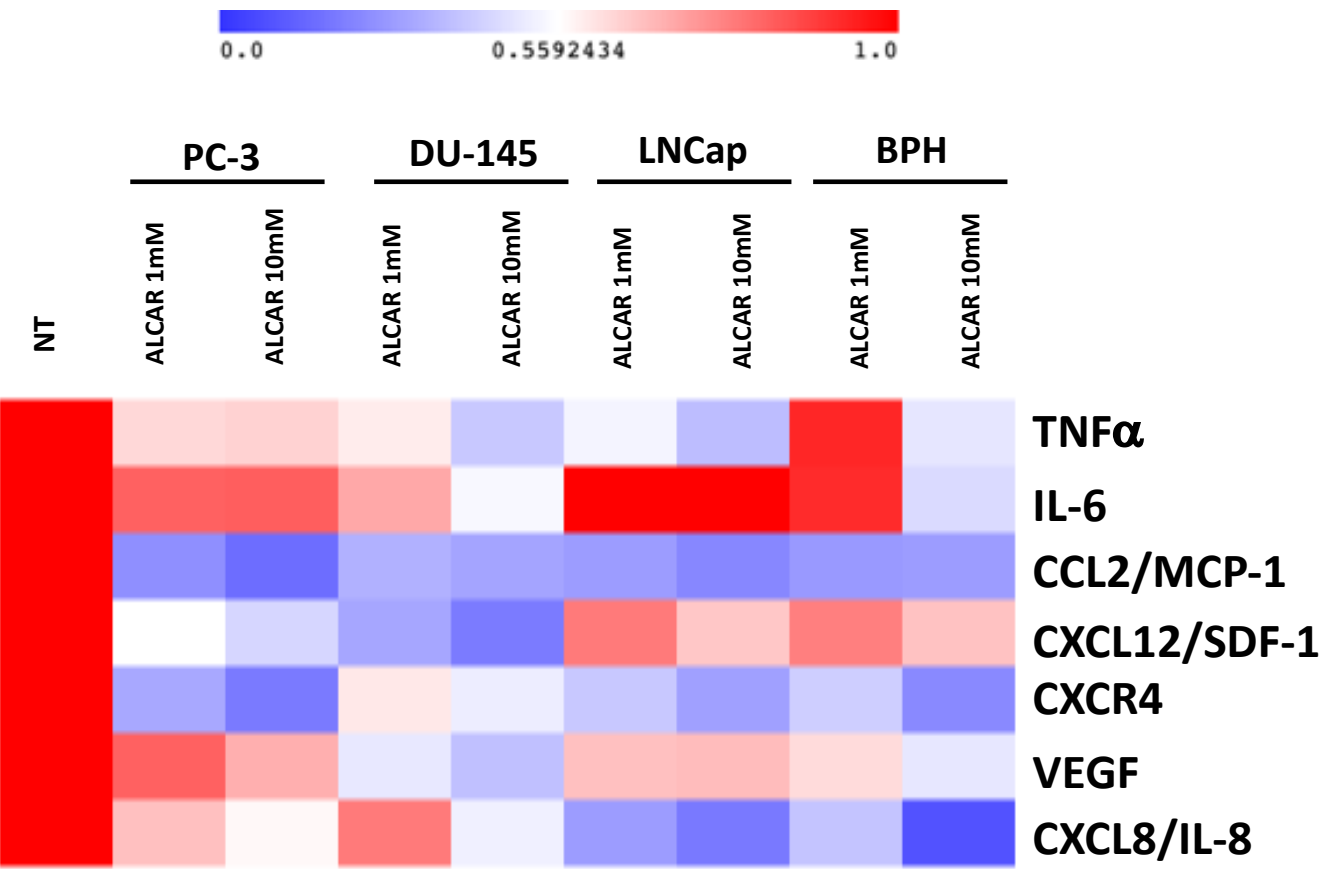

Suppl. Figure 4

A

NT DU-145

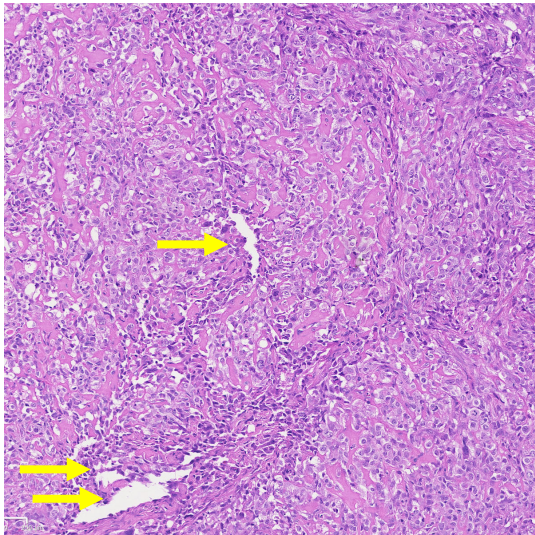

ALCAR DU-145

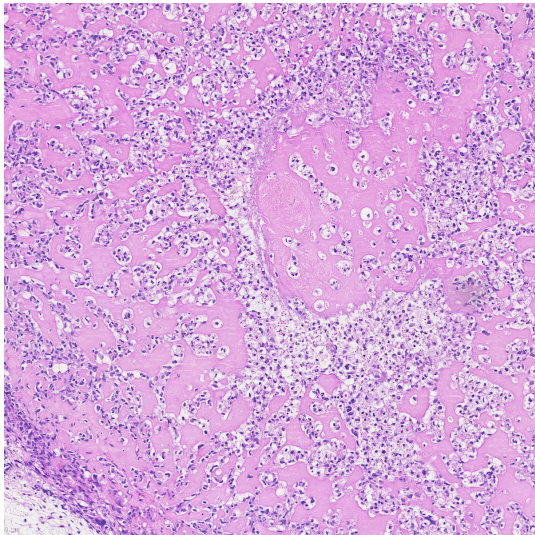

B

NT 22Rv1

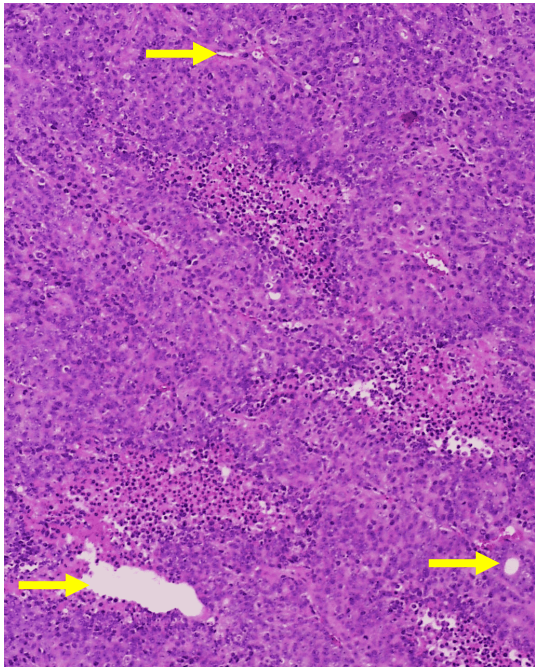

ALCAR 22Rv1

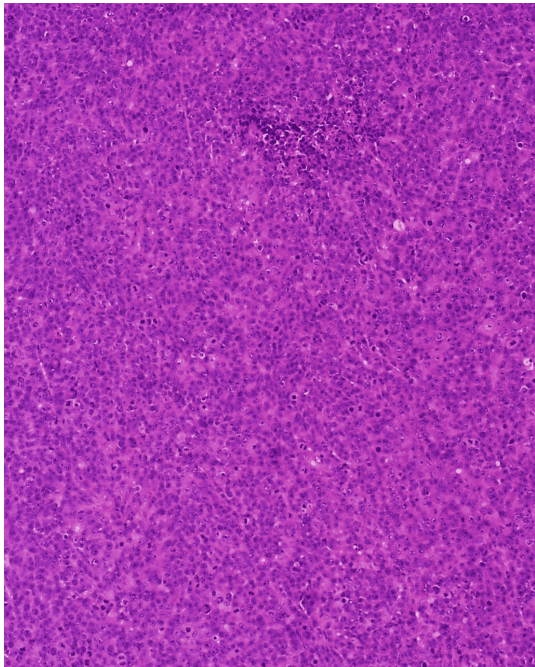

Supplement: Supplementary file 1 — Additional file 1: Figure S1. Effects of ALCAR in the induction of apoptosis inPCa and BPH cell lines. Graphs show the effect of ALCAR (1 and 10 mM, 24 and 48 h) in the induction of apoptosis in PC-3 (A), DU-145 (B), LNCaP (C), BPH (D) cell lines and peripheral blood mononuclear cells (PBMCs) (E) from heathy controls. Stacked graphs refer to different cell state: viable (AnnexinV−PI− cells), early apoptotic (AnnexinV+PI− cells), late apoptotic (AnnexinV+PI+ cells), apoptotic (AnnexinV−PI+ cells). Figure S2. Effects of ALCAR in the induction of cell cycle arrest in PCa and BPH cell lines. Graphs show the effect of ALCAR (1 and 10 mM, 24 and 48 h) on cell cycle in PC-3 (A), DU-145 (B), LNCaP (C), BPH (D) cell lines. Stacked graphs refer to different phase of cell cycle: S phase, Apoptosis, G2/M, G0/G1. No effects on cell cycle was observed. Figure S3. Cytokine/chemokine profiling on PCa and BPH cell lines in response to ALCAR treatment. Representative heatmap for cytokine modulation (CCL2, IL-6, TNF-α, CXCL12, CXCR4, VEGF and CXCL8), as fold-change over non treated cells (NT). Figure S4. Histological analysis of ALCAR treated DU-145 and 22Rv1 xenografts. Haematoxylin/eosin staining (10X magnification) for sections (5 μm) of excised tumours derived from DU-145 and 22Rv1 showing a trend of reduced microvascular density in ALCAR-treated animals (A-F). Arrows indicate vessels. [file 13046_2019_1461_MOESM1_ESM.pdf]
